# Extension of the primary care research object model (PCROM) as clinical research information model (CRIM) for the “learning healthcare system”

**DOI:** 10.1186/s12911-014-0118-2

**Published:** 2014-12-18

**Authors:** Wolfgang Kuchinke, Töresin Karakoyun, Christian Ohmann, Theodoros N Arvanitis, Adel Taweel, Brendan C Delaney, Stuart M Speedie

**Affiliations:** Heinrich-Heine-University, Düsseldorf, Germany; University of Warwick, Coventry, UK; King’s College London, London, UK; University of Minnesota, Minneapolis, USA

## Abstract

**Background:**

Patient data from general practices is already used for many types of epidemiological research and increasingly, primary care systems to facilitate randomized clinical trials. The EU funded project TRANSFoRm aims to create a “Learning Healthcare System” at a European level that is able to support all types of research using primary care data, to recruit patients and follow patients in clinical studies and to improve diagnosis and therapy. The implementation of such a Learning Healthcare System needs an information model for clinical research (CRIM), as an informational backbone to integrate aspects of primary care with clinical trials and database searches.

**Methods:**

Workflow descriptions and corresponding data objects of two clinical use cases (Gastro-Oesophageal Reflux Disease and Type 2 Diabetes) were described in UML activity diagrams. The components of activity diagrams were mapped to information objects of PCROM (Primary Care Research Object Model) and BRIDG (Biomedical Research Integrated Domain Group) and evaluated. The class diagram of PCROM was adapted to comply with workflow descriptions.

**Results:**

The suitability of PCROM, a primary care information model already used for clinical trials, to act as an information model for TRANSFoRm was evaluated and resulted in its extension with 14 new information object types, two extensions of existing objects and the introduction of two new high-ranking concepts (CARE area and ENTRY area). No PCROM component was redundant. Our result illustrates that in primary care based research an important but underestimated portion of research activity takes place in the area of care (e.g. patient consultation, screening, recruitment and response to adverse events). The newly introduced CARE area for care-related research activities accounts for this shift and includes Episode of Care and Encounter as two new basic elements. In the ENTRY area different aspects of data collection were combined, including data semantics for observations, assessment activities, intervention activities and patient reporting to enable case report form (CRF) based data collection combined with decision support.

**Conclusions:**

Research with primary care data needs an extended information model that covers research activities at the care site which are characteristic for primary care based research and the requirements of the complicated data collection processes.

## Background

The increasingly widespread adoption of electronic health records (EHR), as part of the clinical care process has made achievable the reuse of patient data collected during care processes for clinical research. Data derived from the EHR may be useful by providing protocol developers with information about the feasibility of inclusion and exclusion criteria, by simplifying patient recruitment and improving the clinical trial data collection process through populating eCRFs (electronic case report forms) with patient data and avoiding double data collection for clinical care and research. Furthermore, the EHR can play a central role in a system that learns from data collected at the point of care and applies the lessons learned to the improvement of patient care, ensuring the integrity and the quality of the research outcomes, and finally resulting in better diagnosis and treatment innovations. With such rich data, research becomes an important participant in the iterative innovation process known as the “Learning Health Care System (LHS)” [1].

### The learning healthcare system for Europe

The EU funded project TRANSFoRm aims to develop the technology that facilitates a LHS for Europe [2]. Three clinical use cases will drive, evaluate and validate the approach to the Information and Communications Technology (ICT) challenges of embedding diagnostic decision support and clinical trial workflow into the EHR and providing a secure infrastructure for large scale genotype - phenotype studies using primary care data. Important for the implementation of the clinical use cases are several tools, the functional eCRF (an interface for integrating clinical and research data capture into the EHR), mobile eHealth (a web questionnaire for patient reported outcome, ePRO), a Data Quality Tool (to assess the quality of primary care data for research purposes, in terms of completeness, comprehensiveness and validity), and the Query and Data Extraction Workbench (consisting of query authoring for the identification of research subjects based on existing EHR data and a workbench for managing data extraction and linkage for epidemiological studies).

The LHS is the underlying concept of TRANSFoRm; it is driven by an integrated computational infrastructure located at the interface between care and research that supports both patient safety and the conduct of clinical trials. One problem for the implementation of the LHS is that clinical research and clinical care domains are still for the most part disconnected, because each one uses different standards and terminology systems. For TRANSFoRm and the LHS, a semantic framework is therefore necessary to achieve interoperability between clinical research and clinical care domains despite different semantics and data models to support model driven software development. This clinical research information model (CRIM) must therefore be based on the workflow of clinical trials (GORD use case), as well as on the one for cohort studies for Type II Diabetes (T2D) to provide a shared conceptual model for research processes in both domains. In this context, CRIM should set the framework of what is possible in research activities in the care area and structure and restrict the underlying data models of TRANSFoRm. For this purpose, CRIM has to consider the semantic limitations posed on providing interoperability between EHR and eCRF through single source data and PROM (Patient Reported Outcome Measures). In summary, a CRIM is needed for integration of the software components of the LHS that represents concepts, relationships, constraints and operations to specify information requirements relevant to primary care based research. Achieving this informational interoperability and ensuring adherence to common standards are critical to the success of secondary use of care data for research purposes [3].

### Clinical study workflow based modelling

**Figure 1:**
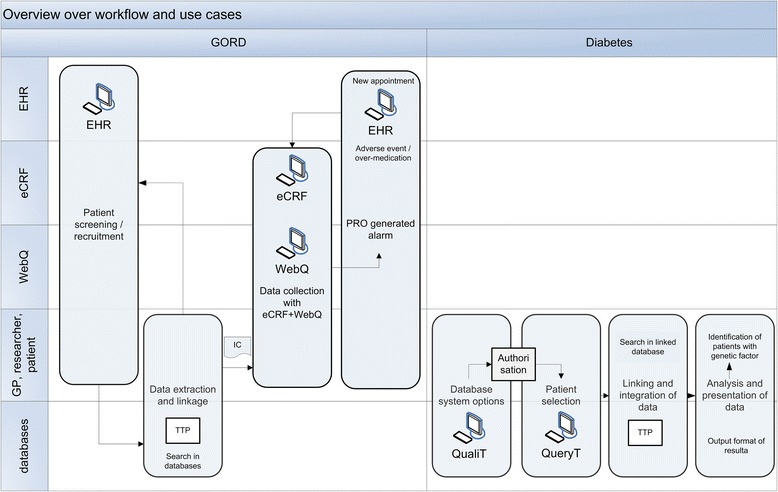
**Overview over the combined workflow, actors and tools of two clinical use cases (GORD and Diabetes).** Actors and tools are indicated in the left frame. Sub-use cases are indicated as grey boxes and connected by arrows to indicate the workflow. EHR = Electronic Health Record, eCRF = electronic Case Report Form, WebQ = Web Questionnaire for Patient Reported Outcome (PRO), GP = General Physician (Family Doctor), QualiT = Quality Tool, QueryT = Query Tool, TTP = Trusted Third Party, IC = Informed Consent.

Our approach is to consider the clinical research workflow more heavily already in the development of information models, since the reuse of care data for clinical research purposes results in complex relationships in the flow of data and information (Figure 1). It has to be considered that organization and information requirements of clinical research performed in a primary care practice differs substantially from that in clinical research settings. For example, EHR systems do not provide for the easy collection of the data required for research or decision support. In addition, the eCRF differs considerably from the data collection form of the EHR. On the other hand, EHR may trigger different types of reminders, invitations, and alerts (e.g. by adverse events) that may be integrated into the research workflow. All these processes imply restrictions on the semantic interoperability and on the software solution developed. Thus, established information models were evaluated to be used as CRIM for both TRANSFoRm research clinical use cases (GORD and T2D) to be employed as information model or as basis for an own information model that is applicable to the LHS.

## Methods

An information model in software engineering is a representation of concepts, relationships, constraints, rules, and operations to specify data semantics for a chosen domain of interest. It can provide sharable, stable, and organized structures of information requirements for the domain context [4]. In this way, CRIM must cover two different domains of interests (care and research) and must represent the complete data flow during clinical research processes and provide the necessary information requirements for tools developed in TRANSFoRm. Our approach consists of applying different levels of modelling and using Use Case driven Object Modelling with UML [5]. We used dynamic structure diagramming of clinical research workflows to map all activities and associated data requirements to the static structures of information model classes. The workflow description and the corresponding information objects of two use cases (Gastro-Oesophageal Reflux Disease, GORD) and Type 2 Diabetes mellitus, T2D) were described in UML activity diagrams using MS Visio™ and Enterprise Architect™ and formed the basis of the evaluation step. Workflow modelling is an established technique for business process re-engineering. In the clinical research domain, the use of workflow modelling is still used only rarely [6]. But often clinical trial modelling in UML can serve as a standard format because it allows the detailed description of research processes in Activity Diagrams to enable process analysis [7]. The information model has to adapt to the requirements of the two very different TRANSFoRm research use cases spanning a broad area of medical research. Whereas GORD is designed as a randomized controlled trial (RCT) with compliance to good clinical practice (GCP), T2D is a non-interventional study based on existing data using a case–control design and database research. Nonetheless, both use cases are linked by a number of identical actors. First, patient (consults GP, flagged as possible study participant, obtains study pseudonym), second, GP (searches for eligible patients, checks inclusion/exclusion criteria) and third, researcher (defines criteria). The process descriptions cover the use of eCRF embedded in the EHR and web questionnaires (WebQ) for data collection as well as a data quality tool and a Query Workbench.

Making use of the activity models and use cases, we then proceeded to find a suitable information model for the implementation of the defined tasks employing TRANSFoRm tools consisting of objects and/or classes and their associations. The purpose of the resulting model was to act as CRIM that specifies the necessary information objects, their relationships and associated activities that are required to fully support the development of TRANSFoRm tools. All activity objects of the workflows were defined and characterized according to their data requirements and information needs and mapped to the concepts of established information models (PCROM [8], BRIDG [9], SDM [10], CTOM [11,12], openEHR [13], HL7 RM [14]).

In a first step, it was analysed what information is required at each stage of the research process for the activities to be performed and which details of the workflow are relevant for the corresponding information flow. The relevance of identified information object types and differences in assignments were identified, and it was considered if existing concepts and connections in established information models were appropriate, in terms of classes in the primary care domain or the research domain. In a second step, objects were added to extend PCROM and aligned and evaluated with the workflow, to verify if they exhibit an improved representation. The resulting model was compared with the other information models to confirm that an improvement in the representation was achieved. Through the modelling process we developed a complementary set of information objects which have a conceptual and relational connection with the workflow activities at the overlapping area between care and research (e.g. alerts (alarms), appointments, reminders, web questionnaire). Based on the results of this evaluation, PCROM was extended by the addition of several new concepts.

## Results

The best mapping results were achieved with PCROM (Table 1) and it was decided to use PCROM as basis for the development of CRIM. Comparison of PCROM to BRIDG found a significant overlap of concepts but also several areas important to research that were either not yet represented or represented quite differently in BRIDG [9]. The comparison between PCROM and BRIDG showed that PCROM is an easier representation of RCT than BRIDG and can easier support the interoperability needs arising from the development of electronic clinical trials systems.

**Table 1:** **Domain and information objects identified in two clinical use case workflows (GORD and Diabetes) and their relation to PCROM and BRIDG**

**No**	**Class/domain objects**	**Comment/description**	**PCROM objects**	**BRIDG objects**
**1**	AE	Is result of assessment, assessment activity	AE, AEreport	DefinedAdverseEvent, AdverseEvent, (AdverseEventActionTakenRelationship, AdverseEventOutcomeAssessment, AdverseEventOutcomeResult, AdverseEventSeriousness)
**2**	Alert (alarm)	Is event (defined notification), process activity / study event (e.g. for AE event), alert is reaction to assessment		DefinedNotification,
**3**	Appointment	Is related to care (e.g. GP visit)		
**4**	Close-out visit	(also for follow-up visit) is a study event in a study, consultation	Study event	StudyActivity (DefinedActivity)
**5**	Consultation	Is related to care		
**6**	CRF	Belongs to data model, display as form covering observation activity, assessment activity and intervention activity		Document (DocumentAuthor)
**7**	Eligible patient	Is person, the result of eligibility search is a process activity	Potential participant (singular)	
**8**	Examination		Examination (is observational activity)	PlannedActivity, DefinedActivity
**9**	GP	Is person or role (family doctor, health care provider)	GP investigator	HealthCareProvider
**10**	GP visit	Is related to care		
**11**	Inclusion and exclusion criteria		EligibilityCriteria	DefinedEligibilityCriterion, DefinedInclusionCriterion, DefinedExclusionCriterion
**12**	Informed consent	Is a activity, potential participant consents (is a relation)	Consented as relationship	StudySubject, StudyProtocol (not directly dispayed)
**13**	Invitation of patient	Is related to care	Notification	
**14**	Medicinal product	Is product, (medicinal intervention as a new class, covers “product”)	Intervention (generic), (medical intervention)	StudyAgent (Product, Drug)
**15**	Patient initiation	Potential participant, intervention	Intervention activity	DefinedProcedure
**16**	Patient recruitment	Is process activity, see: eligible patient, participation is result	Participant (as result)	StudySubject (as result)
**17**	Randomisation	Is a study procedure, process activity	Allocation	RandomizationBookEntry
**18**	Reminder	Is event (scheduled notification), process activity /study event		DefinedNotification, PlannedNotification, NotificationReceiver
**19**	Research question	Is part of study protocol	Study purpose	StudyObjective
**20**	Responsiveness	Is assessment of PPI responsiveness = assessment results, assessment activity	Assessment results, assessment activity	StudyActivity
**21**	Search	(Is in GORD the eligibility search)		
**22**	Symptoms	Result of an observational activity	Observation activity result	DefinedObservation result
**23**	Web questionnaire	Is active process, e.g. PRO, observation is different from the activity of data input; here it is observation activity		DefinedObservation
**24**	Class / Domain Objects Use Case Diabetes	Comment / Description	PCROM objects	BRIDG objects
**25**	TTP	Is an organization	Organization	
**26**	Eligible patient	Is a person, is also result of eligibility search and therefore a process activity	Potential participant (singular)	
**27**	Inclusion and exclusion criteria		EligibilityCriteria	DefinedEligibilityCriterion, DefinedInclusionCriterion, DefinedExclusionCriterion
**28**	Informed consent	Is a activity, the potential participant consents is a relation	Consented as relationship	StudySubject, StudyProtocol (not directly displayed)
**29**	Patient	Is a person	Patient	Subject
**30**	Prescription	Is a process activity in medical care (also in T2D: search in prescription data base, belongs to data model)	Activity	Activity
**31**	Researcher	Is a person	Investigator, StudyActor	QualifiedPerson, ResearchStaff, ResearchOrganization
**32**	Search	Is a process activity in T2D		
**33**	Symptoms	Result of an observational activity	Observation activity result	DefinedObservation result
**34**	Data extraction	Is a process activity		
**35**	Database access	Is a process activity		
**36**	Linkage	Is a process activity		
**37**	Patient selection	Is a process activity		
**38**	Remote analysis	Is a process activity		
**39**	Genetic risk factor		Assessment Result	
**40**	Dynamic Data Discovery Service	Belongs to data model (software concept)		
**41**	Query Tool	Belongs to data model (software concept)		

Class/Domain Objects are mapped to corresponding information objects in PCROM and BRIDG. Objects that could not be mapped are classified as belonging to the “data model” or being a “process activity”. These objects were not considered for the information model but are relevant for the data model of TRANSFoRm. Objects that were used for evaluation but belonged to the data model instead of the information model are not shown in the table.

### Coverage of randomized clinical trials, patient recruitment and cohort studies (database research)

**Figure 2:**
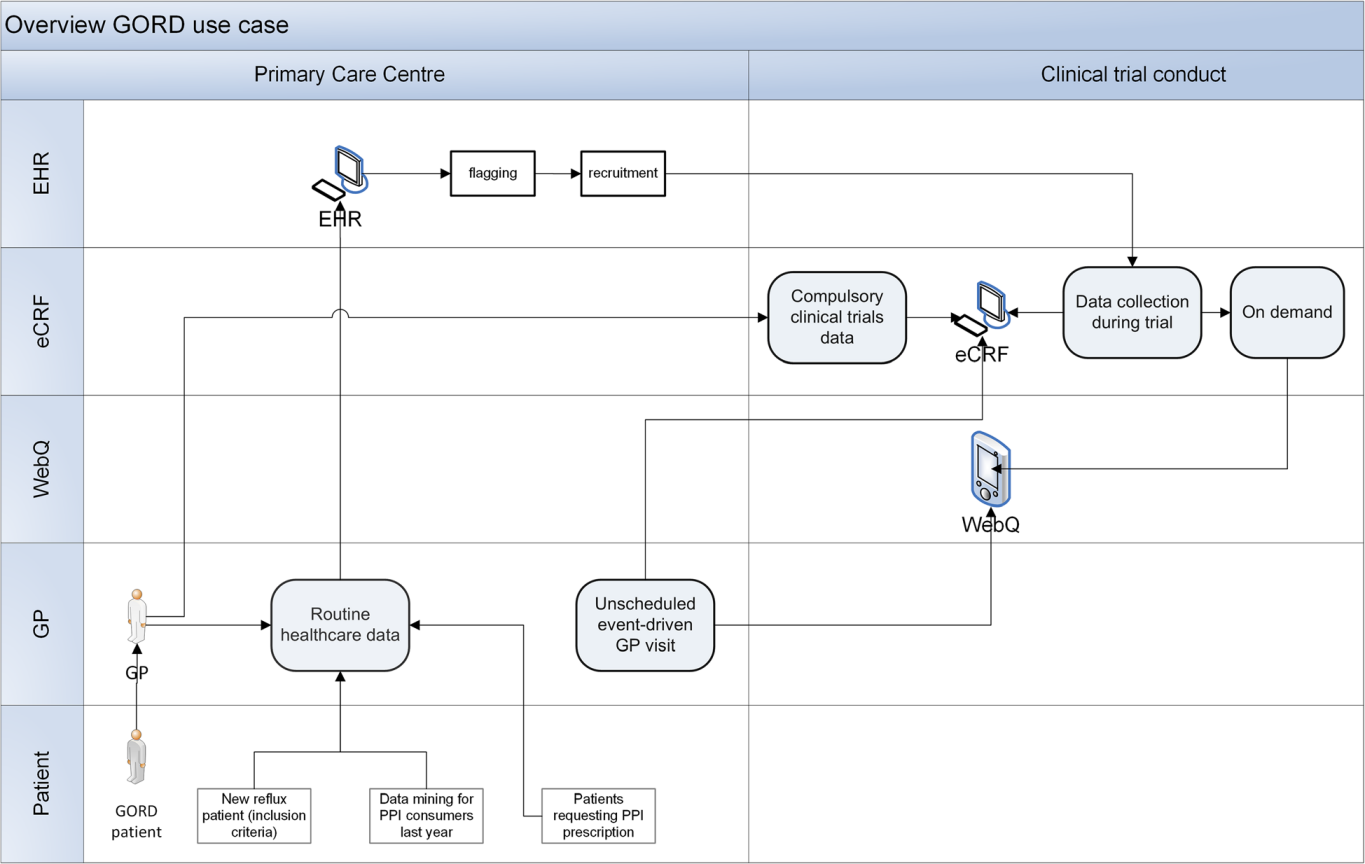
**Workflow of the GORD clinical use case with three different types of data collection.** Data is collected by “EHR, eCRF and WebQ” and used in a randomized clinical trial.

The general approach for the comparison of the different information models is to use activity diagrams to display the workflow of two very different clinical research use cases (GORD and T2D) that cover a large area of possible clinical research activities. GORD represents a RCT comparing the effectiveness of continuous vs. on-demand use of acid suppression on symptoms and quality of life (QoL) with a 1 year follow-up period. GORD also contains an event-initiated assessment of QoL information in subjects with reflux disease and the identification and recruitment of eligible patients (Figure 1). Potential study participants will be identified by different methods: firstly, by data mining in EHR for PPI (proton pump inhibitor) consumers during the previous year; secondly, by identifying patients requesting PPI prescriptions during an appointment and thirdly, during appointments of the GP with new reflux patients. The use case description already points to the importance of the GP appointment in the initiation of the research study. In addition, GORD employs different types of data collection methods at several time points (Figure 2). During the study entry visit and at month 3, 6, 9 and 12, information about PPI consumption is collected by web questionnaires and/or EHR (structured data collection). In addition, event driven data collection by eCRF is performed (e.g. in case of adverse events (AE)).

**Figure 3:**
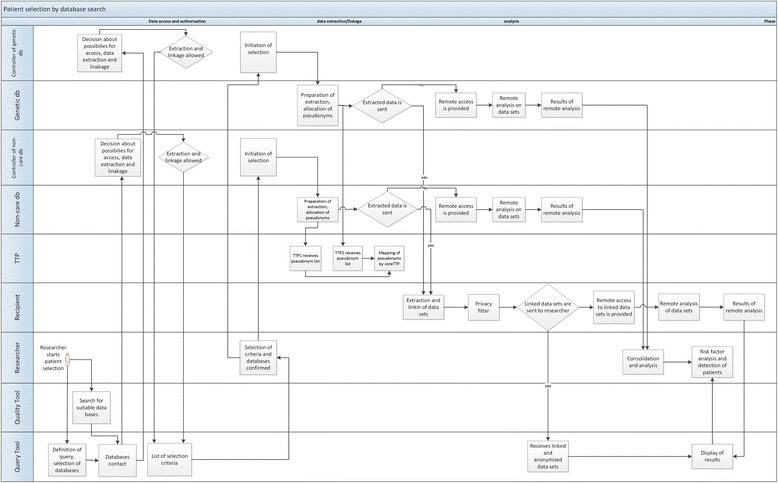
**Workflow of the Diabetes use case.** Actors and tools are indicated in the left frame. The use case shows how databases are searched for patient selection and for clinical trial participation. db = database, TTP = Trusted Third Party.

In contrast, the Diabetes use case tries to determine if selected single nucleotide polymorphisms (SNPs) are associated with the development of T2D complications in populations, and especially, if an association exists between SNPs and variations in the drug response to oral anti-diabetics. To answer this research question, selection of patients as well as data extraction from various databases has to be performed. This includes the selection of T2D patients from genomic databases, and the selection of patients with a specific medication history from primary care databases. The extracted information includes medication, SNPs, glycosylated haemoglobin (HbA1c), time since diagnosis, etc. But to compare these data from different databases, it is necessary that data are linked at the individual patient level. Data extraction and data linkage is done by the Query workbench, which is used for the selection and flagging of patients in different databases and the associated data extraction process. Figure 3 among others the part of the workflow that deals with patient selection by database search. The authority for primary care data and genetic data is represented by the “data controller”, a defined role of the EU Data Privacy Directive [15], who is responsible for the data in the corresponding database. The data controller decides who has access to the data and what can be done with the data (“data processing”).

### Considering the complex activities of primary care based research

**Figure 4:**
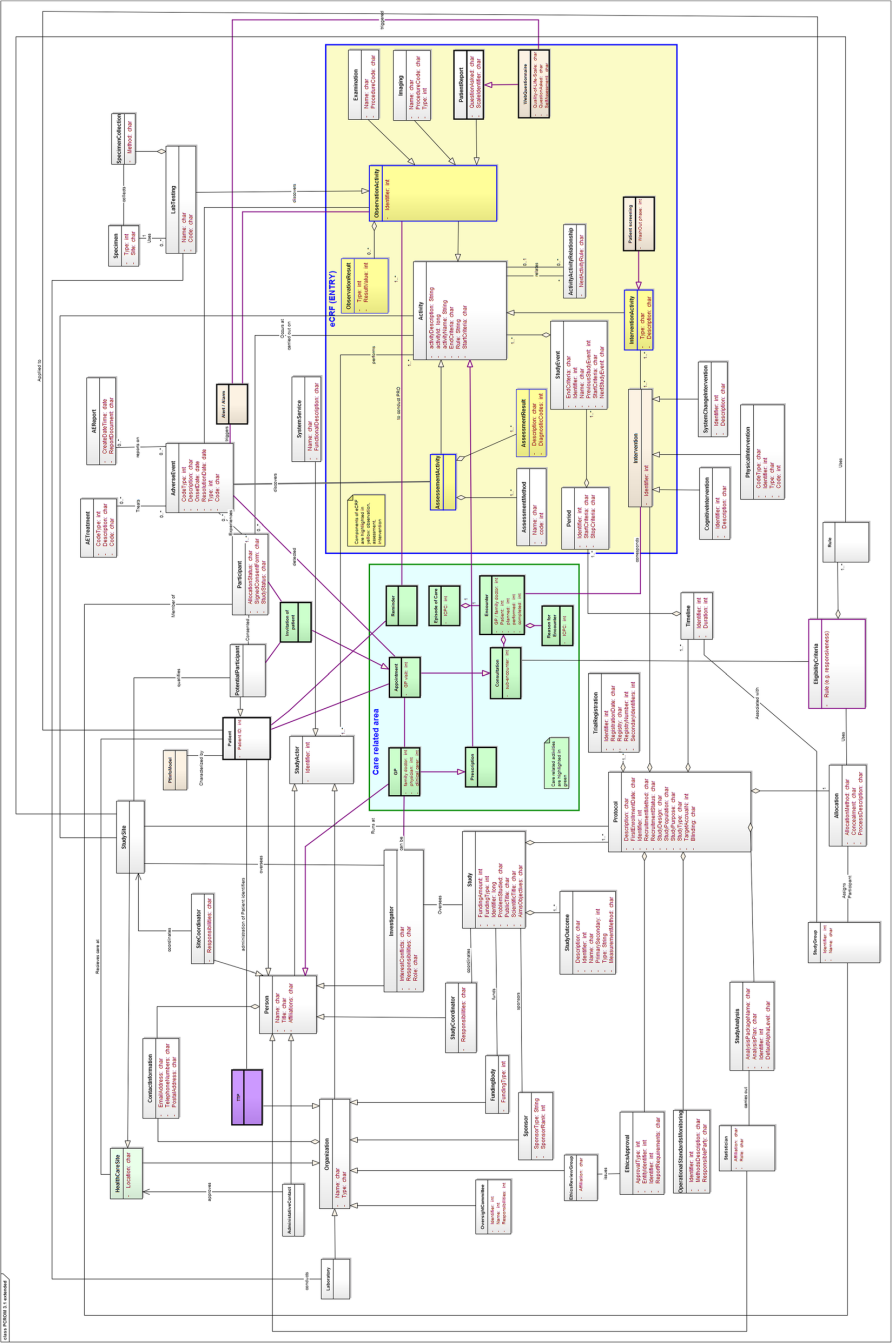
**Extended PCROM (Clinical Research Information Model).** Shown is the extended PCROM as UML class diagram. The “Care related area” (green) and the eCRF (ENTRY) area (yellow) are framed by as bold line. New objects are indicated by bold framing in purple and in some cases in additional colouring. Objects with a tight connection to the CARE or ENTRY area are shown in the corresponding colour. For example, the class “Invitation of patient” and HealthCareSite are in green, because they belong to the CARE area. In the ENTRY area classes that constitute the concept of a functional eCRF are shown in yellow (e.g. ObservationActivity, ObservationResult, AssessmentActivity). New objects not belonging to CARE or ENTRY areas are coloured in purple (e.g. Alert/Alarm, TTP). New relationships introduced are indicated by bold arrows in purple.

At the basic level, the GP (as care giver and as researcher) and the patient become Study Actors in CRIM, describing any entity that plays a role in the clinical research process (Figure 4). Data collection is supported by the actors EHR, eCRF (eSource enabled [16]) and WebQ. Primary care data is available in health care databases, prescription databases and genetic databases. In addition, a Trusted Third Party (TTP) and a recipient database are necessary to link data sets in a way compliant with data protection and privacy requirements. The TRANSFoRm Query Tool and Quality tool enable the identification of suitable databases and the querying in different primary care databases (Figure 1). In contrast, the GORD use case consists of several sub-use cases that detail different components of the RCT, including patient screening and recruitment, data extraction, data collection, and PROM (Patient Reported Outcome) generated alarm.

### Extension of PCROM to obtain a CRIM for both use cases

The evaluation of different information models showed that PCROM is able to represent the information needs of research activities in both use cases nearly completely, but with two exceptions: the areas of data collection and of GP/patient interaction are not covered adequately. For example, before a patient is enrolled in a clinical trial and becomes a study participant, considerable interactions between GP and patient, including Encounter, Appointment and Consultations triggered by Reminders and Alerts, can take place. To account for these missing activities, we developed a complementary set of information objects which have a conceptual and relational representation of workflow activities at this overlapping area between care and research (Table 2). Several of these new concepts have to do with specific interactions of the GP (family doctor) with the patient, like examination, diagnosis, invitation of patient, patient initiation, prescription). Therefore the central concept of Encounter as part of an Episode of Care (International Classification for Primary Care, ICPC [17]) was introduced into CRIM. The concepts of AssessmentResult (diagnosis) and Intervention (corresponds to process) are already part of PCROM. Encounters as such are characterized by reason for encounter, diagnosis (corresponds to AsessmentResult) and procedure (corresponds to intervention).

**Table 2:** **Objects added to the extended PCROM**

**No.**	**Object**	**Connections**
**New information objects**		
**1**	Reminder	Patient, ObservationActivity
**2**	Alert / Alarm	AdverseEvent, ObservationActivity, WebQuestionnaire
**3**	TTP	Organization, Patient
**4**	Web questionnaire	PatientReport, AlertAlarm
**5**	GP	Person, Investigator, Appointment, Prescription
**6**	Invitation of patient	PotentialParticipant, Participant, Appointment
**7**	Prescription	GP, Activity
**8**	Appointment (incl. GP visit)	Patient, Invitation of patient, Adverse Event, Consultation
**9**	Encounter	Episode of Care, Reason for Encounter, Intervention
**10**	Reason for Encounter	Encounter
**11**	Consultation	Encounter, Appointment, EligibilityCriteria
**12**	Episode of Care	Encounter
**13**	PatientScreening	InterventionActivity
**14**	PatientReport	ObservationalActivity, WebQuestionnaire
**High-level areas**		
**15**	eCRF (ENTRY)	ObservationalActivity, ObservationalResult, AssessmentActivity, AssessmentResult, InterventionActivity
**16**	CARE related	GP, Prescription, Appointment, Consultation, Reminder, Episode of Care, Encounter, Reason for Encounter
**Extension of existing information objects**		
**17**	EligibilityCriteria	Rule (e.g. responsiveness)
**18**	Patient	Patient ID

On the other hand, several concepts of the workflow description are only part of a data model and have little importance for the information model (e.g. data check, database controller, data controller, EHR, genetic database, care database). The difference between both models exists in the fact that the main purpose of an information model is to display objects at a conceptual level, independent of any specific implementation or data management protocol [18], whereas data models are defined at a lower level of abstraction and include specific details of data structure and protocol-specific constructs [18]. In particular, many of the objects representing the T2D use case relate more strongly to the data model than to the information model. These objects were not further regarded for the evaluation.

The result of our model verification and mappings between activity diagrams and information objects was that only 14 unique concepts/objects (with 2 areas and 2 extensions of existing objects) were sufficient to extend PCROM (Figure 4) and to satisfy the information needs of both research use cases: reminder, alert/alarm, TTP, web questionnaire, GP (family doctor), invitation of patient, prescription, appointment (incl. GP visit), Encounter (Reason for Encounter), consultation, Episode of Care, patient screening, patient report (complete list in Table 2). In principle, many relevant information objects are already presented in PCROM (e.g. Eligible patients, Intervention, Protocol, StudyCoordinator and Researcher).

### Introduction of high-level units to account for the complexities of data collection and patient contact

**Figure 5:**
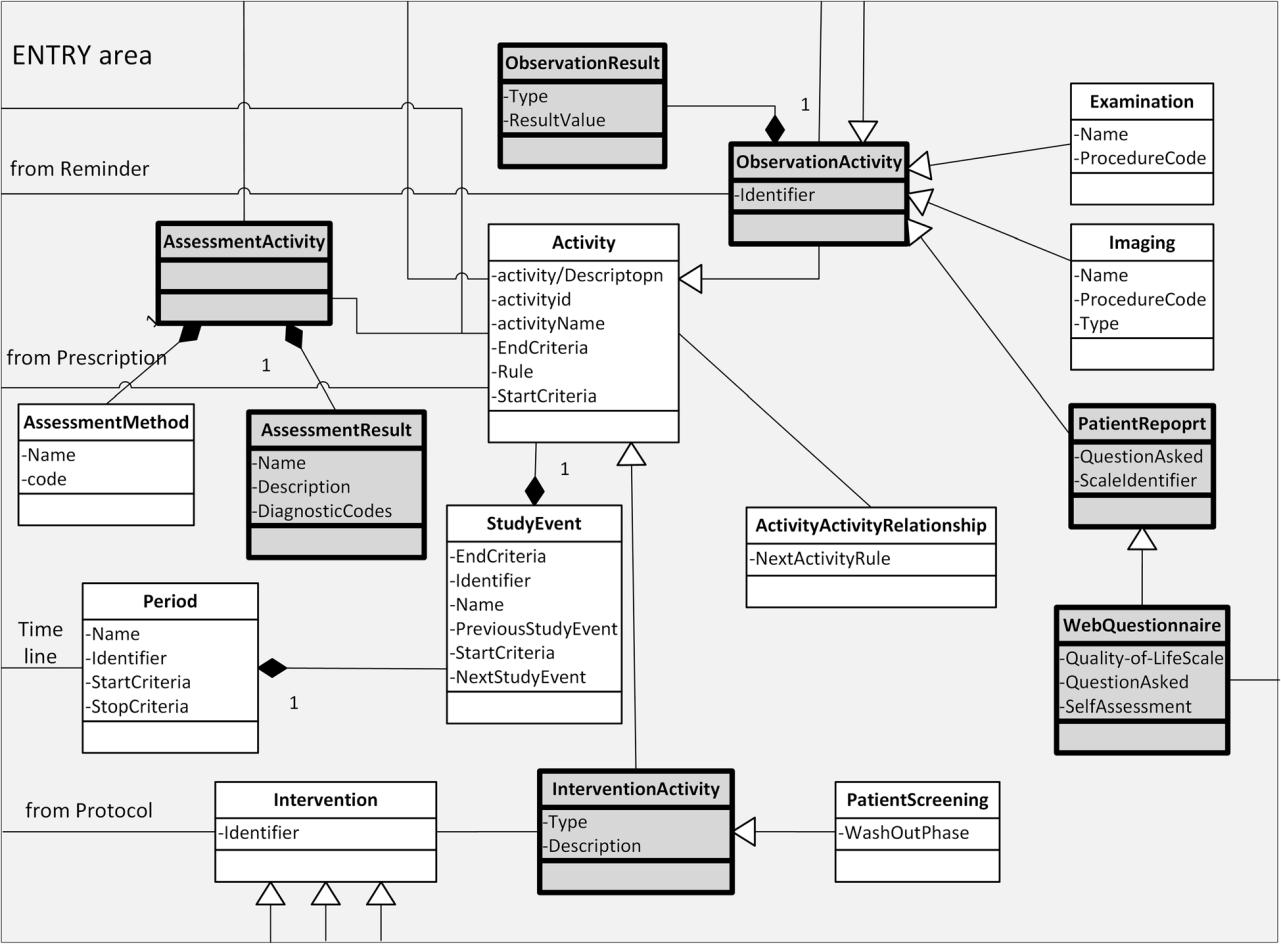
**Detailed depiction of the “ENTRY Area (eCRF)” (light grey) of the extended PCROM.** Dark grey objects form part of the eCRF level of the area.

In addition to the added 14 information objects, two new high-level units were introduced. The unit ENTRY (Figure 5) combines the objects Intervention, AssessmentResult, ObservationActivity, ObservationResult and AssessmentActivity. This unit represents the central role of the eCRF as a heterogeneous information object. The novel object WebQuestionnaire was added and linked to ObservationActivity (for ePRO and QoL). PatientScreening (including the wash out phase) was added and linked to InterventionActivity. In addition, outside and independent of the care area a TTP was added as a new organization; it includes PatientIdentifier.

**Figure 6:**
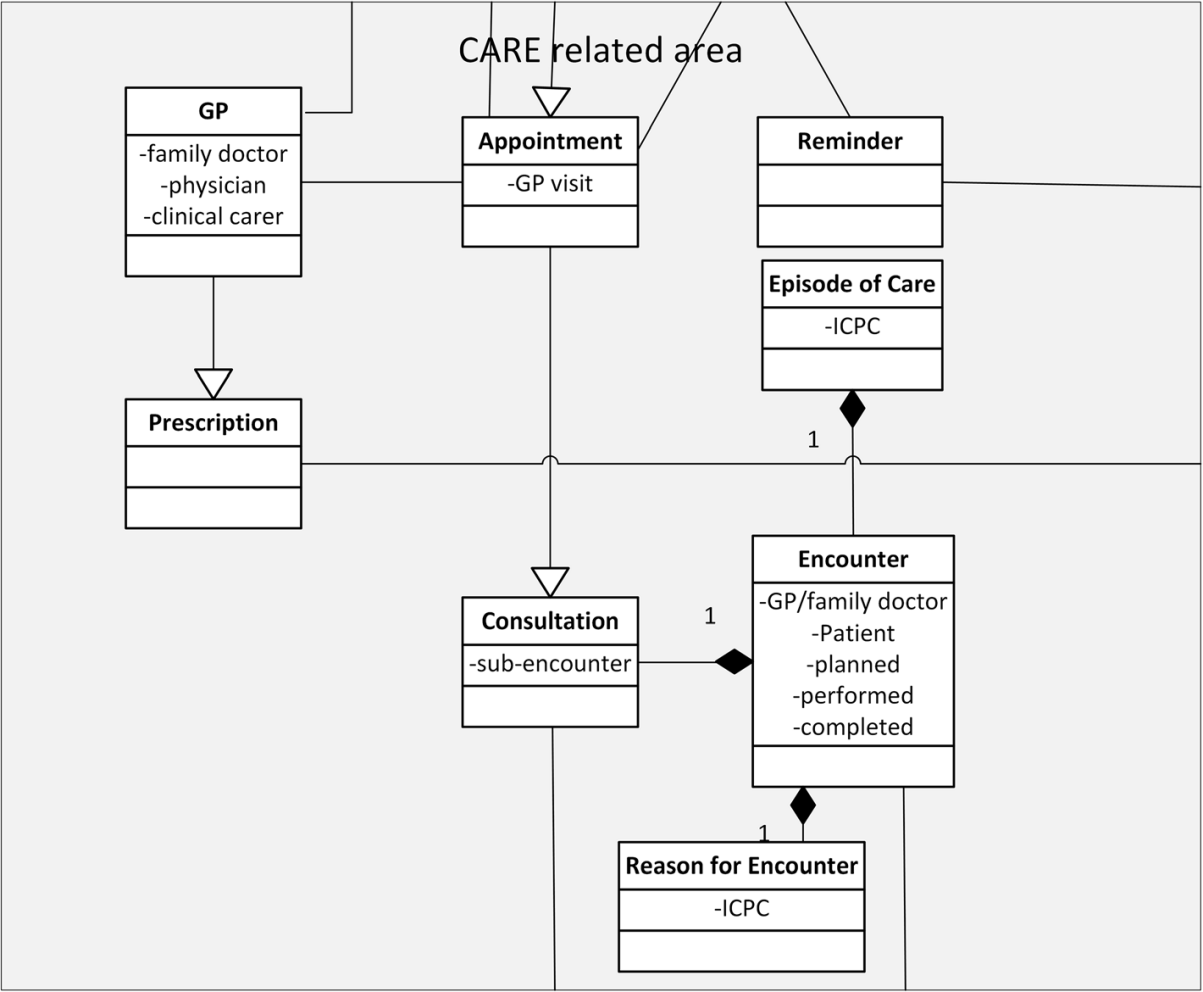
**Detailed depiction of the “CARE related area” (light grey) of the extended PCROM.** Encounter, Reason for Encounter and Episode of Care are integrated. ICPC = The International Classification of Primary Care.

To account for the information flow during patient/participant interaction with the GP, a second high level unit was introduced: the CARE related area (Figure 6). It joins all objects that are located at the area overlapping care and research activities. Here patient identification and screening (e.g. appointment, reminder) takes place and it includes: Reminder, GP, Invitation of patients, Prescription, Appointment and Consultation). The object EligibilityCriteria was extended by Responsiveness. The GP is a Person and connects with an Appointment to the Patient. Appointment can be care and research related as well as adverse event (AE) related. Thus, it connects with the Patient, and the Participant through Invitation, but also to AdverseEvent. The GP (family doctor) represents following main activities: Appointment, Consultation, Encounter and Prescription. The object of Encounter was introduced to have a high-ranking concept that allows for different forms of interactions between patient and GP. Because Encounter describes the professional interchange between a patient and a GP that is related to the concept of Episode of Care, the Episode of Care considers a collection of Encounters (visits) that are related to a condition or treatment plan. But appointments with the GP can also be initiated by alerts or invitations. For example, the GP can receive a reminder that is study relevant to invite a patient to complete an ePRO questionnaire for a study. Because the GP embodies the double role of treating physician and researcher, relations have to connect the GP as care giver to the research area. For example, an appointment caused by an adverse event becomes an unscheduled study event. In this context, the gap from the potential participant to the study participant was connected by “Invitation of patient” and Consultation was connected with Encounter and “Episode of Care”. An appointment of the patient with the GP can be a consultation (which is an encounter with a “Reason for Encounter”) and is connected to EligibilityCriteria outside the CARE area. Eligibility criteria decide about the participation of a patient in a clinical trial. Using these organizing concepts from ICPC, our information model increases the clarity of terminology in this complex area of overlap between care and research and it connects the activities in the care area to the information structure of clinical research represented in PCROM.

Data collection in clinical trials is done with the eCRF that is a special form designed to collect high-quality information. In our use case, the eCRF plays a central role as integration hub (functional eCRF) bringing together information from EHR, study database and WebQ (Figure 2). It is represented in our model as an additional area (ENTRY), covering observational aspects and assessment aspects (Figure 5). The rational for this area lies in the complexity of the data collection process in clinical trials containing besides the actual data collection process, aspects of Observations, Assessment, Intervention, StudyActivity, Examination and StudyEvent. In our model, the ENTRY area collects these aspects and combines them on a higher level. For this reason, the WebQuestionnaire for QoL assessment is connected to PatientReport; thus the WebQuestionnaire becomes an instance of PatientReport. Because the original PCROM only contained the concept of Interview, we added the more general object of PatientReport allowing the integration of different data collection methods (e.g. web questionnaire) (Figure 4). The added Alarm/Alert is an automatic ObservationActivity. The TTP links to Patient by association; and the characteristic Pat ID becomes an attribute of Patient whereas the TTP becomes a repository for Pat IDs.

## Discussion

There is a movement from a static representation used for information modeling to a more dynamic representation that is able to consider quite complicated workflow processes associated with primary care based and other forms of research. In this sense, our use cases are characterised by an area of patient screening and recruitment that has decision points and forking of processes, as well as the querying of primary care data, prescription and genetic databases that shows decision points and parallel processes. Because the primary care domain is so complicated and diverse, it has been doubted that a single information model may be possible [19]. But, by extending the PCROM information model we obtained a CRIM able to consider that in TRANSFoRm the EHR and CRF are not only data input interfaces but can also trigger the generation of reminders and initiate the distribution of invitations and alerts (in case of adverse events or overdosing) at defined time points on the study timeline (e.g. visits and appointments). With CRIM we were able to include care and research aspects, covering clinical trials, patient recruitment and database research, in a single information model.

We based our modeling efforts on PCROM, because, in contrast to the other information models, its basic structure seemed suitable to extensions based on research processes: the main axis of PCROM describes the information objects for participant – activity (observational activity, assessment activity, intervention activity) – study event. Forty-five percent of PCROM objects have been mapped to BRIDG, 37% were differed in class and/or subclass assignment, and 18% did not map at all [8]. We did not consider the Clinical Trials Ontology (OCR) [20] as basis for our information model; because the focus of this ontology is not on research processes but on study design (containing descriptors of study design and a categorization of studies by their design descriptors) and research analysis. For example, OCR has borrowed epochs, arms, and activities from the BRIDG model.

A comparison of PCROM with existing healthcare and clinical trial related information models (CTOM [12], BRIDG [9], HL7 RIM [14], openEHR [13], and CDISC (SDM) [10]) showed their drawbacks in relation to PCROM. Table 3 summarizes features that were important for our review. Compared to PCROM these information models turned out to be either too limited in scope or too complicated to represent both use cases. Nonetheless, analysis of these information models yielded valuable insights to ensure comprehensiveness and correctness of interpretation of information objects for the verification and extension of PCROM.

**Table 3:** **Comparison of key characteristics of reviewed information models**

**Aspect**	**CTOM version 1.3 (2007)**	**PCROM version 2.0**	**BRIDG (version 3.02) PR sub-domain**	**CDISC SDM version 1.0**	**HL7 RIM 2.41**	**EHR RM, 1.02**	**Comment**
**Basic concepts**	Activity, study event	Activity, study event	Study activity	Activity, study event	Entity, Role, Participation, Acts	Care_entry, admin_entry, instruction, activity	Differences with respect to relation between activity and study event
**Representation of structural concepts of clinical trials**	Study time point		Epoch	Epoch, cell, segment	A medical record is a record of each of the individual actions that make up the diagnosis, treatment and care of a patient	Through observation, history, event	Differences in granularity, in some cases indirect representation
**Link to study protocol**	From activity via study participant via site to protocol	From study event via period via timeline to protocol	From study activity to sub-protocol version	Protocol elements exist within categories: structure, workflow, timing			Different concepts (link via patient, standard elements of protocol)
**Representation of care aspects**	Health care site, health care site participant	Health care site, patient	Health care provider (subject)		SubstanceAdminis-tration, PatientEncounter, DiagnosticImage	Care_entry, event_con-text	Represented, except for CDISC SDM

PR = Protocol Representation, SDM = Study Design Model.

In detail, CTOM [12] is very much oriented towards cancer research; including special classes for the method of tumor detection (e.g. imaging). It is ruled by three basic classes: activity, observation and assessment. Activity is connected with procedures (e.g. surgery, imaging), observation is connected with histopathology, clinical results and assessment is connected with AE, diagnosis, etc. We studied the activity - observation - assessment axis for our model.

The CDISC Study Design Model (SDM) [10] describes XML elements, their attributes, and their relationships that are relevant for study design. The terms elements and objects are used to reference XML constructs, or alternatively, to reference objects and properties of those objects. Study execution is outside the scope of this model; it separates distinct aspects of study design into sub-components: structure (arms, cells, segments, activities, etc.), workflow (decision points, branches, etc.) and timing (time points of activities, usually relative to other structural elements of the study). We studied the workflow and timing components to compare them with our workflow.

The BRIDG Model [9,21] is an instance of a Domain Analysis Model (DAM) for protocol-driven research and as a DAM it contains no semantics that is based on a particular “solution space” but is associated with regulatory artefacts (e.g. data, organizations, resources, rules, processes, concerned with pharmacological, physiological, or psychological effects of drugs and procedure, and associated regulatory artefacts).

Since the scope of BRIDG is extensive, several sub-domain models are provided: Adverse Event Sub-Domain (covers safety related activities, like detection, evaluation, follow-up and reporting), Common Sub-Domain (the semantics that are common to all (or most) of the other sub-domains and might even be common to any healthcare-related domain analysis model, including people, organizations, places and materials), Protocol Representation Sub-Domain (covers the planning and design of a research protocol), Regulatory Sub-Domain (covers the creation and review of regulatory submissions), Study Conduct Sub-Domain (covers the execution of a research study). The recent BRIDG version (v3) includes even a statistical sub-domain. This subdivision seems pragmatic and potentially applicable to the situation in the TRANSFoRm use cases. BRIDG defines: (1) Defined Activities that are the characterization of a kind of activity, i.e. they define “what” an activity is; (2) Planned Activities are the association of defined activities to a particular study. This association also includes the characterization of the timing of activities; (3) Scheduled Activities are the instantiation of a planned activity for each subject of a study; (4) Performed Activities represent the execution of activities for actual subjects on a study and the results of those activities. For our modelling, we concentrated on protocol representation and therefore considered only Defined Activity and Planned Activity. The subdivision of the model into Defined Activities, Planned Activities, Scheduled Activities and Performed Activities makes the model too complicated to be used for our efforts; especially, because our use cases are not limited to regulatory protocol-driven research. As a consequence, we used BRIDG in the background of our model to map PCROM objects to BRIDG and to use BRIDG objects as possible resource to assess and modify PCROM.

Because HL7 RIM [14,22] is the most comprehensive information model for all healthcare aspects; we included it in our evaluation of the usefulness for research representation. Because of its breadth, RIM tends to use generic classes, attribute and association names that are not necessarily domain-friendly. It expresses the way data content, needed in a specific clinical or administrative context within the healthcare domain, can interoperate syntactically and semantically. RIM is composed of six back bone classes: Act, Participation, Entity, Role, Relationship and Role link. Four core concepts play an important role according to their associations: Entities: physical units such as things, human beings, organisations, groups; Roles: time bound named functions for an entity, e.g. healthcare provider; Acts: intentional actions, e.g. healthcare encounter, referral, intervention, etc.; Participations: links between an act and a role. As example to depict the complexity of this information model, it is sufficient to look at how Observation, the basis for collecting patient data, is represented. In RIM for Adverse Event the ObservationEvent is connected via subjectOf4 with Document (universal). For the Performed Observation the ObservationEvent is connected through trigger2 with PerformedObservationResult. These are quite complex relations and it is unclear what the relation between ObservationEvent and the collection of the observation could be for describing our own use cases.

Though highly complex, there exists a mapping between BRIDG and RIM. The version 3 of BRIDG contains cross-referencing between BRIDG UML and HL7-RIM [23]. The comparison shows that from BRIDG to RIM some research specific semantics seems to get lost. For example, all three BRIDG elements PerformedClinicalInterpretation, PerformedClinicalResult and PerformedDiagnosis are represented in RIM only by ObservationEventResult. A Performed Observation consists of several ObservationEvents, but is also connected to SubstanceAdministrationEvent, ProcedureEvent, etc. In summary, RIM aims to provide a single set of reference semantics that can be leveraged across all healthcare domains; this may be the reason that BRIDG semantics cannot easily be mapped to HL7 RIM and that the RIM representation seems to be too far away from research processes as its focus is primarily health services and clinical care.

The Clinical Document Architecture (CDA) [24] was created as a XML document based standard that specifies the structure and object that can include text, images, sounds, and other multimedia content for clinical documents. In principle, CDA may be used to support data collection for research purposes. From the information model point of view, the Refined Message Information Models (R-MIM) are used to model specific scenarios within HL7 v3; R-MIM contains only those classes and attributes required to compose a specific set of messages or documents. In general, subdomains are derived from RIM through well-defined rules to create R-MIMs and are used to model specific case scenarios within the HL7 V3 standard. Each R-MIM is a subset of the D-MIM, and a D-MIM is a subset of the RIM. The R-MIM contains only those classes, attributes, and associations required to compose the specific set of messages or documents and the CDA R-MIM is the most popular one, which is used for the exchange of clinical documents. The ClinicalDocument is a component of classes that describe the document structure (NonXMLBody, StructuredBody, Section, …). From our point of view the concept of the clinicalStatement choice box, containing classes for observation and encounter, is interesting. The clinical statement entries used in CDA contain Encounter, as an interaction between patient and care provider for the purpose of providing healthcare services. The clinical statement modeling (entry) in CDA utilizes the HL7 V3 RIM and Data Type R1 specifications to enable semantic interoperability. As a result of our evaluation, CDA was regarded as being too high a level to be useful for the clinical use cases which require specific information objects that are able to characterize a patient, including for example genomic data.

The EHR Information Model (openEHR) [13] is a model of an interoperable EHR from the RM/ODP (Reference Model of Open Distributed Processing) viewpoint, providing a framework for the standardization of open distributed processing (ODP) and defining a logical EHR information architecture. In principle, openEHR implementations can easily generate ISO EN 13606 [25] communication extracts, because ISO EN 13606 represents specifications for the exchange of “EHR Extracts” [26]. Technically it may resemble a simplified version of the openEHR reference model. For example, the different ENTRY types of openEHR are mapped to a single ENTRY type in ISO EN 13606. The openEHR reference model (EHR RM) [27] is complemented by additional information models (e.g. Demographic IM, EHR Extract IM, Data Structures IM) and clinical models that are formalised models of health domains content. For data capture a Context Model of Recording is provided. This is necessary, because the openEHR model takes into account the importance of context in clinical activities. For example, the context of data entry is considered, in which the information generated by a “healthcare event”, containing “clinical statements”, is added. All information created in the openEHR health record is expressed as an instance of a class in the entry package, containing the ENTRY class. For data collection, an ENTRY instance is a single “clinical statement”, and may be a single short narrative phrase, but may also contain data. In terms of clinical content, the ENTRY classes are important elements in the EHR Information Model, since they define the semantics of all the information in the record. Context sensitive data entry is a concept that inspired our creation of the ENTRY area of CRIM. In CRIM we have to distinguish and integrate semantically between data entry in the care setting with the one in the research setting. In the end, we introduced an overarching ENTRY concept (see modelling) and added it to the extended PCROM. In the care setting, data collection is linked to the EHR, the interacting physician and alerts/reminders generated by the EHR; whereas in the research setting data collection is done by eCRFs, the physician as investigator and with patient data that is pseudonymized.

PCROM [8] was developed for the types of research projects and clinical trials typically carried out in primary care settings [8]. Its sub-modules contain organisations, people, and systems with a focus on the entities actually involved in a clinical trial. The Trial Information submodel focuses on classes that describe the nature of the trial with the core concept of a Study. PCROM doesn’t need any other subdivision, like the distinction of planned vs. performed activities we find in BRIDG or classes related to a software application and its associated documents. On the other hand, BRIDG makes more frequent use of the concept of a relationship class (from HL7/RIM) [8], to describe relationships between or among multiple instances of a particular class. PCROM has placed this type of relationship at the level of an Activity. In summary, PCROM takes a much simpler view of the event flow within a study than does BRIDG and considers strongly trial related activities. Thus, the PCROM approach offers greater flexibility in portraying sequences of events than other models and is easier adaptable to the workflow processes of the two TRANSFoRm clinical use cases.

After the evaluation of different information models, PCROM made the most convincing case to act as a backbone for CRIM. Only three main classes: Protocol, Study and Activity exist and all other elements link to these core elements. For example, Activity is linked via StudyEvent and Period/Timeline with Protocol, mirroring the way that the study protocol determines the conduct of a trial. The usability of this concept has been confirmed by the fact that PCROM served as component of the electronic Primary Care Research Network (ePCRN) [28]. Although, depicting satisfactorily the structure of a RCT, PCROM lacks in two areas: the screening and recruiting of patients and the incorporation of different forms of data collection based on the EHR as central component, triggering PROM and using single source data collection. Because we extended PCROM by only a small number of new objects and concepts to close these gaps, it retains the simplicity and clearness that distinguishes PCROM from other models and at the same time maintains a tight relationship with BRIDG.

The extended PCROM (CRIM) covers all relevant concepts from the overlapping area between care and research, like web questionnaire, invitation of patient, prescription, appointment (incl. GP visit), patient screening, consultation, etc. and includes them in a structure already optimised of clinical trials. On the negative side, CRIM concepts are rather generic from the viewpoint of the interoperability needs of data models, with classes that are not specific enough for database focused research creating a gap between the semantic requirements defined by CRIM and the requirements of the different databases involved. Our approach of a rather generic CRIM developed gradually during the TRANSFoRm project and is based on the realisation that in research based on primary care data, a multitude of different data structures, data models and standards on the level of different EHRs and care data databases has to be adapted. To be complete in this regard would overstretch any CRIM. Thus, we developed CRIM to be software application agnostic (e.g. EHR and care databases) and to use a hierarchy of two different models in TRANSFoRm. On the first level (semantic interoperability), CRIM informs the design of eCRFs, WebQ and the study structure. Here, it links to CDISC ODM and SDM and provides restrictions to data integration. On the second level (data interoperability/integration), the Clinical Data Integration Model (CDIM) [29] presents the data view, enabling access to the data in different databases. Because CRIM is based strictly on the workflow of clinical research, it informs and restricts on what can be done in the research process and how to build the corresponding clinical trial or research study. This division into two models was necessary, because for the T2D use case of database focused research CRIM is less necessary than for the GORD use case, and the emphasis in the first case is on CDIM that incorporates models of different databases. CRIM doesn’t need to link to different trial systems and database models and can delegate this task to CDIM. Nonetheless, CRIM integrates on a higher level the semantic restrictions of RCT and database focused research, a necessity because both use cases overlap in the area of identifying patients for study recruitment by using searches in primary care databases.

## Conclusions

A workflow-oriented information model allows many user needs and preferences to be incorporated into system development and implementation. For CRIM, we developed a complementary set of information objects which have a conceptual and relational connection with workflow activities at the overlapping area between care and research (e.g. alerts/alarms, appointments, reminders, web questionnaire). The developed extended PCROM (CRIM) is the information model for all aspects of clinical research in TRANSFoRm and it will constitute the informative basis and part of the integrated computational infrastructure of TRANSFoRm’s “Learning Healthcare System” which provides a hub between care and research. It can be employed, when research is done not only according to conventional RCT, but also with involvement of the care area and care databases. This means the physician or researcher is able to search EHR data for eligible patients, to employ a screening and recruitment period or to use alerts and reminders generated by the EHR to inform the data collection process. Because of the heterogeneity of data models employed by primary care databases, CRIM must be complemented on the interoperability level with a data integration model (CDIM) that covers different data structures.

Our model supports the concept of the physician as researcher. Thereby, it builds upon a new relationship between physician and patient in that practicing physicians should be prepared to respond to requests from patients about research, advising patients about clinical trials, and thereby enhancing the physician-patient relationship [30]. To support investigator initiated trials the mindset of inquiry and observation should be encouraged in physicians [31,32]; this should be done in addition to support the development of an adequate IT infrastructure for primary care research to move towards evidence-based learning.

## Abbreviations

AE: Adverse event
BRIDG: Biomedical research integrated domain group
CDA: Clinical document architecture
CDISC: Clinical data interchange standards consortium
CRF: Case report form
CRIM: Clinical research information model
CTODS: Clinical trials object data system (further development of CTOM)
CTOM: Clinical trial object model
DAM: Domain analysis model
D-MIM: Domain message information model
EHR: Electronic health records
EHR RM open: EHR reference model
ePCRN: Electronic primary care research network
eSource: Electronic source data
GORD: Gastro-oesophageal reflux disease
GP: General physician (family doctor)
HbA1c: Glycosylated haemoglobin
HL7 RIM: Health level 7 reference information model
ICPC: International classification for primary care
ICT: Information and communications technology
OCR: Clinical trials ontology
openEHR: Open standard specification for health data in electronic health records
Pat ID: Patient identification number
PCROM: Primary care research object model
PPI: Proton pump inhibitor
PRO: Patient reported outcome
PROM: Patient reported outcome measures
QoL: Quality of life
RCT: Randomized controlled trial
R-MIMs: Refined message information models
RM-ODP: Reference model of open distributed processing
SDM: Study design model (CDISC)
SNP: Single nucleotide polymorphisms
T2D: Diabetes type 2
TTP: Trusted third party
TRANSFoRm: Translational research and patient safety in Europe
UML: Unified modeling language
WebQ: Web questionnaire
XML: Extensible markup language
